# Medfly-*Wolbachia* symbiosis: genotype x genotype interactions determine host’s life history traits under mass rearing conditions

**DOI:** 10.1186/s12896-019-0586-7

**Published:** 2019-12-18

**Authors:** Georgios A. Kyritsis, Antonios A. Augustinos, Ioannis Livadaras, Carlos Cáceres, Kostas Bourtzis, Nikos T. Papadopoulos

**Affiliations:** 1 Insect Pest Control Laboratory, Joint FAO/IAEA Programme of Nuclear Techniques in Food and Agriculture, A-1400 Vienna, Austria; 2 Laboratory of Entomology and Agricultural Zoology, Department of Agriculture Crop Production and Rural Environment, University of Thessaly, Phytokou St., 38446 N, Ionia Magnisia, Greece; 3 Foundation for Research and Technology - Hellas (FORTH) Institute of Molecular Biology and Biotechnology, FORTH, Nikolaou Plastira 100, Vassilika Vouton, GR - 700 13 Heraklion, Crete Greece

**Keywords:** *Wolbachia*, *Ceratitis capitata*, Symbiosis, Genotype, Fitness, Sterile insect technique, Incompatible insect technique, Tephritidae, Fruit flies

## Abstract

**Background:**

*Wolbachia pipientis* is a widespread, obligatory intracellular and maternally inherited bacterium, that induces a wide range of reproductive alterations to its hosts. Cytoplasmic Incompatibility (CI) is causing embryonic lethality, the most common of them. Despite that *Wolbachia*-borne sterility has been proposed as an environmental friendly pest control method (Incompatible Insect Technique, IIT) since 1970s, the fact that *Wolbachia* modifies important fitness components of its hosts sets severe barriers to IIT implementation. Mass rearing of Mediterranean fruit fly, *Ceratitis capitata* (medfly), is highly optimized given that this pest is a model species regarding the implementation of another sterility based pest control method, the Sterile Insect Technique (SIT). We used the medfly-*Wolbachia* symbiotic association, as a model system, to study the effect of two different *Wolbachia* strains, on the life history traits of 2 *C. capitata* lines with different genomic background.

**Results:**

*Wolbachia* effects are regulated by both *C. capitata* genetic background and the *Wolbachia* strain. *Wolbachia* infection reduces fertility rates in both *C. capitata* genetic backgrounds and shortens the pre-pupa developmental duration in the GSS strain. On the other hand, regardless of the strain of *Wolbachia* (*w*Cer2, *w*Cer4) infection does not affect either the sex ratio or the longevity of adults. *w*Cer4 infection imposed a reduction in females’ fecundity but *w*Cer2 did not. Male mating competitiveness, adults flight ability and longevity under water and food deprivation were affected by both the genetic background of medfly and the strain of *Wolbachia* (genotype by genotype interaction).

**Conclusion:**

*Wolbachia* infection could alter important life history traits of mass-reared *C. capitata* lines and therefore the response of each genotype on the *Wolbachia* infection should be considered toward ensuring the productivity of the *Wolbachia*-infected insects under mass-rearing conditions.

## Introduction

*Wolbachia pipientis*, an obligatory intracellular maternally transmitted alpha proteobacterium, was first identified in *Culex pipiens* in 1936 [1]. Recent studies have estimated that more than 40% of the terrestrial arthropod species have evolved symbiotic relationships with *Wolbachia* [2]. This extensively wide host spectrum stimulated abundant research aiming to establish factors explaining the evolutionary success of *Wolbachia*, which could be highly attributed to its ability to manipulate biological functions of its hosts in a way that assures both the bacterium and the host continuity through generations.

The broad array of reproductive manipulations used by *Wolbachia* include the induction of parthenogenesis, feminization, male killing, and Cytoplasmic Incompatibility (CI). CI is the most common *Wolbachia*-induced, reproductive phenomenon that results in embryonic lethality when a *Wolbachia* infected male mates with an uninfected female or a female that carries a different *Wolbachia* strain [3–5]. Considering the sterility induction to uninfected populations, the idea of exploiting the *Wolbachia* infection towards implementing insect pest control has been proposed since early ‘70s as an environmental friendly pest control method (Incompatible Insect Technique-IIT method) [6, 7]. “Dictation” of reproduction (sterility induction in this case) is not a stand-alone phenomenon and the presence of *Wolbachia* is often accompanied with a broad spectrum of responses in host organisms which have not been fully elucidated. Such effects may favor or limit the potential for IIT implementation in case of positive and negative effects respectively.

Unravelling the effects of *Wolbachia* infection on insects’ biology has become an intriguing experimental field over the last few decades. The *Wolbachia*-insect endosymbiotic relationship has been correlated with a wide range of effects (negative, neutral or positive) on major biological parameters, such as fecundity, fertility, mating behaviour and adult lifespan [8–20]. There are often contradictory experimental results regarding effects of *Wolbachia* on insect host life history and behavior, even among different populations of the same species, that are attributed to i) the “dynamic” nature of the *Wolbachia*-host symbiotic relationship, and ii) the determinant role of both insect and *Wolbachia* genomic backgrounds on the expression of a given biological modification. For example, Weeks et al. (2007) pointed out that the fecundity disadvantage imposed by *w*Ri to *D. simulans* evolved into a fecundity benefit in less than 20 years, whereas among the mosquitoes’ genus *Aedes, Wolbachia* infection has been associated either with negative or neutral effects on adults’ longevity [12, 17, 18]. Additionally, studies on insect behavior conducted mainly on *Drosophila* spp. demonstrated that *Wolbachia* could contribute to reproductive isolation between infected and uninfected populations [19, 21–24].

The Mediterranean fruit fly (medfly), *Ceratitis capitata* (Wiedeman) (Diptera: Tephritidae), is one of the most damaging pests to fruit production worldwide. The broad range of host plants, the wide geographical distribution, combined with multivoltinism makes the control of this pest challenging and large scale Area-Wide Integrated Pest Management (AW-IPM) projects the most appropriate strategy to achieve sound control [25, 26]. Often the implementation of the Sterile Insect Technique (SIT), successfully implemented in several parts of the globe over the last three decades, consists the main element of many AW-IPM projects. In contrast to medfly, the implementation of SIT for other target species is facing quite challenging barriers. Some of the major obstacles are (a) the poor productivity of large scale rearing, and (b) the inadequate performance of the mass-reared insects under field conditions [27, 28]. Both aforementioned limiting factors are probably stemmed from the absence of suitable insect strains that are fully adapted to mass-rearing conditions and the currently used rearing protocols. *Wolbachia* infection could potentially abate some of the aforementioned productivity and biological quality concerns through the modifications imposed on hosts’ biology. In addition, CI expression in a novel host could set the scene for a combined application of irradiation and symbiont-based, sterility induced, pest control methods (combined SIT and IIT approach) [29–31].

The transfer of *Wolbachia* to medfly, a non-host species (but see [32]) that constitutes the most optimized pest in terms of the Area-Wide SIT control, constitutes an interesting biological “framework” to clarify pending symbiotic-related inquires at both basic and applied level. Zabalou et al. (2004, 2009) [33, 34] set the stage for such an experimental approach by using embryonic cytoplasmic injections and managed to establish three *Wolbachia*-infected medfly lines carrying two different, *Rhagoletis cerasi* derived, *Wolbachia* strains: the “S10.3” carrying the *w*Cer4 and the “88.6” and “56S2 Genetic Sexing Strain (GSS)” carrying the *w*Cer2 bacterium strain. A series of laboratory experiments conducted to evaluate the artificially infected lines, showed that all three *Wolbachia*-infected medfly lines exhibit considerable stability, inducing 100% of cytoplasmic incompatibility in the novel host [33, 34]. Additional laboratory studies revealed that *Wolbachia* infection seems to reduce medfly fertility, fecundity and lifespan whereas also shorten the developmental duration [35]. However, it is not known whether the *Wolbachia* impact on *C. capitata* biological traits has “evolved” over time or conferred additional alterations. Moreover, none of the previous studies evaluated *Wolbachia* effects on *C. capitata* behaviour nor under mass rearing conditions.

Given the impact that *Wolbachia* infections may have on the life history traits of a host species, and particularly on its rearing efficiency and male mating competitiveness, in the present study we used the medfly-*Wolbachia* symbiotic associations, as a model system, to study the effect of two different *Wolbachia* strains, on the life history traits of two medfly lines with different genomic background. Exploiting this system under small scale mass-rearing conditions is expected to pave the ground for utilizing *Wolbachia* symbiosis as a tool to enhance Sterile Insect Technique approaches. Our findings are discussed in the context of the evolution of symbiotic association, the effects of *Wolbachia* on novel hosts, as well as from an applied perspective since *Wolbachia* is part of the tool kit towards the development of environmental friendly methods for population control of insect pest species of agricultural, veterinary and human health importance.

## Materials and methods

### Flies used

*Laboratory lines*: We used five Mediterranean fruit fly laboratory lines (see Additional file 1). The *Wolbachia* uninfected lines: (a) “Benakeio”, a laboratory line that has been maintained under laboratory conditions for more that 30 years, and (b) the Vienna 8 medfly genetic sexing strain (“Vienna 8 GSS” hereafter) reconstructed in 2012, carrying the D53 inversion and two mutations that allow male-only releases (the temperature sensitive lethal mutation (tsl) eliminates females after egg exposure to specific temperatures and the white pupae colour mutation (wp) assigns different colour to male and female pupae) [36]. And, the *Wolbachia* infected lines: (a) ‘88.6’, a transinfected Benakeio line carrying the *w*Cer2 *Wolbachia* strain, (b) ‘S10.3’, a transinfected Benakeio line carrying the *w*Cer4 *Wolbachia* strain, and (c) ‘56S2 GSS’, a transinfected Vienna 8 GSS line carrying the *w*Cer2 *Wolbachia* strain. Both *Wolbachia* strains (*w*Cer2 and *w*Cer4) are naturally found in field populations of *Rhagoletis cerasi*, which was the donor species for the establishment of the *Wolbachia*-infected medfly lines [33, 34], (from now on any reference to medfly genetic background will be noted by capital letters, VIENNA 8 GSS or BENAKEIO, whereas any reference to each one of the five medfly lines will be noted by small letters, Vienna 8 GSS, Benakeio, 56S2 GSS, 88.6, S10.3).

*Wildish flies:* Male mating competitiveness of the five medfly laboratory lines were tested against the F1 generation of a medfly population collected from Volos (Central Greece), from field infested bitter oranges (pupae were sent to the FAO/IAEA Insect Pest Control Laboratory, that reared for one generation).

### Rearing conditions

Experiments were conducted at the FAO/IAEA Insect Pest Control Laboratory, Seibersdorf Austria, from June 2013 to February 2014. Adults from the laboratory strains were reared in fine mesh covered, rectagular cages (200x180x20 cm, ≈200,000 flies in each) provided with water and adult diet consisting of yeast hydrolysate (MP Biochemicals) and sugar at a 1:3 ratio, respectively [37]. Females oviposited through the fine mesh, and eggs dropped (and are collected) in trays containing water (placed below the mesh). Eggs were placed on carrot diet where the larval development took place [38]. The wild adult females recovered from Greece were allowed to oviposit on bananas (the banana peel was pierced with a needle in order to facilitate oviposition), where larvae developed. All medfly colonies were kept at 22 °C and 65 ± 2% RH and a photoperiod of 14 L:10D with the light phase starting at 07:30.

### Medfly and *Wolbachia* infection status

*Samples collection and DNA extraction*: Prior to experiments, 20 adults (10 males and 10 females) were collected upon emergence and immediately placed at -20 °C. DNA was isolated using the Qiagen DNeasy kit (Qiagen, Valencia, CA), following the manufacturer’s instructions.

*PCR based Wolbachia screening*: the *Wolbachia* presence was tested for all individuals by amplifying a *Wolbachia-*specific 16S *rRNA* gene fragment of about 438 bp using the *Wolbachia* specific primers *wsp*ecF and *wsp*ecR [39].

*PCR based screening for “wCer” strains*: All individuals that were found *Wolbachia-*positive were screened for the presence of different *Wolbachia* strains (*w*Cer1 to *w*Cer5) using the previously reported *wsp* gene-based PCR [40].

Three individuals from each of the infected strains were sequenced for the five Multilocus sequencing typing (MLST) genes (*gatB, coxA, hcpA, fbpA* and *ftsZ*), to verify beyond doubt the presence of the expected *w*Cer strain (*w*Cer2 or *w*Cer4). Amplicons were amplified using the primers and PCR conditions described in the *Wolbachia* MLST database (https://pubmlst.org/wolbachia/info/protocols.shtml).

### Effect of *Wolbachia* on demographic traits

We used the rearing-cages described above (see 2.) to collect the biological material for the demographic experiments. Eggs laid within a period of 24 h were placed on strips of black filter paper on a wet sponge infused with 3^o^/_oo_, Propionic acid to prevent fungal growth. Twenty-four hours after the egg collection, 1000 eggs were transferred into a Petri dish (radius x height: 70 × 15 mm), containing 150 g of a carrot larval diet [38]. Petri dishes were placed over sawdust, the larvae popped out of the diet to pupate and the pupae were collected by sieving the sawdust. We performed at least three replicates with 980–1000 eggs each, for each one of the medfly lines tested. Egg hatch, pupation, and adult emergence were recorded once a day at 11:00. Immature development took place under controlled temperature, humidity and illumination (22 °C, 65 ± 2% RH, 14 L:10D). In order to determine effects on adult lifespan and fecundity, upon emergence one female and two males were placed in 40 cm^3^ rectangular cages, having ample access to adult diet and water. At least 10 cages were tested for each of the five medfly lines. One side of the cages was covered with fine mesh, which was used by females to lay eggs through the fine mesh on a piece of moist black filter paper placed below the mesh. The eggs were counted under a stereoscope and the cages were inspected for dead flies at 12:00 daily throughout their lifespan. The cages were kept under constant environmental conditions (22 °C, 65 ± 2% RH, 14 L:10D) until the end of the experiment.

### Assessing *Wolbachia* effect on males’ mating competitiveness

Male mating competitiveness of the five medfly lines (Vienna 8 GSS, 56S2 GSS, Benakeio, 88.6 and S10.3) was assessed against wild males for wild females (F1 progenies of a field collected population). The experiment was conducted in standard field cages (2.0 × 1.6 × 1.9 m) (placed in a glasshouse) housing one potted *Citrus sinensis* Osbeck (Rutaceae) tree, under controlled temperature and humidity (26 ± 1 °C, 45–55% RH respectively). Soon after emergence (within 24 h) flies were sorted by sex and kept in cylindrical Plexiglas cages (≈100 flies in a volume of 6.5 L). Water and a standard adult diet were supplied ad libitum. Male mating competitiveness was tested at the age of 5–7 days, against 11–13 days old wild males, for wild females of same age. The day before conducting the mating tests, adult males were marked on the thorax with a non-toxic dye (red or yellow colour) in order to distinguish the type of the male that achieves copulation. The colour used for the wild and treated males was alternated between treatments during different replications to exclude any possible effect on females’ mate choice. On the day of the test, males were released into a field cage at 07:30 and were allowed to occupy positions on foliage and perform the typical sexual performance before the release of females that took place at 09:00. At least two observations per hour were made until the termination of the mating test at 15:00. Mating couples were removed and placed into transparent plastic vials where they were kept until the end of copulation. Twenty five females and 50 males (25 wild and 25 of each individual population tested) were released in each field cage. The field cages were randomly allocated to treatments. We performed at least five replicates (field cages) for each medfly line.

### Effect of *Wolbachia* on flight ability

The procedure described in detail in [41] was followed to assess effects of *Wolbachia* infection on adult flight ability. Two days before emergence, 100 pupae were placed within a ring of paper, which was centered in the bottom of a Petri dish (100 × 15 mm). One black Plexiglass tube (89 mm diameter, 10 mm high) was placed over a Petri dish. The inside of the tube was lightly coated with unscented talcum powder to prevent the flies from walking out. We recorded the number of individuals that could fly out of the tube. Five replicates (100 pupae each) were set up for each medfly line tested. All tests were conducted in a controlled environment (26 °C and 65% RH, 14 L:10D and 1500 lx light intensity over the tubes).

### Effect of *Wolbachia* on adult longevity under food and water deprivation

Within 4 h of adult emergence (07:30–11:30 am), 30 males and 30 females were placed in a large Petri dish (150 × 15 mm) with a mesh-covered window in the lid and a hole of approximately 15 mm in the center of the lid. All dishes were kept in the dark at 26 °C and 65% RH, until the death of the last fly. Dead flies were sorted by sex, counted and removed from the Petri dishes twice a day (every 12 h; at 19:30 pm and 07:30 am). We performed five replications (Petri dishes) for each medfly line tested.

### Statistical analysis

Data analyses were performed using the SPSS v20.0 (SPSS Inc., Chicago, IL, U.S.A.). The effect of medfly genetic background (VIENNA 8 GSS and BENAKEIO) and *Wolbachia* infection on all biological parameters studied in this paper was determined by analyzing the data of the two uninfected medfly lines (Vienna 8 GSS and Benakeio) and the two infected with *w*Cer2 medfly lines (88.6, Vienna 8 GSS). The effect of the *Wolbachia* strain (*w*Cer2 and *w*Cer4) on the same biological parameters was determined by analyzing the three laboratory populations (Benakeio, 88.6, S10.3).

Binary logistic regression analysis was used to infer the effects of medfly genetic background and *Wolbachia* infection on egg hatch and the survival rates during larval and pupal stages. Chi-square tests, followed by the Bonferroni correction for pairwise comparisons, were used to infer the effects of *Wolbachia* strain on egg hatch, larval and pupal survival rates.

Cox regression analysis was used to determine the effects of medfly genetic background and *Wolbachia* infection on the developmental duration of the immature stages (pre-pupa duration) [42]. The effect of *Wolbachia* strain on the developmental duration of the immature stages was exlored by Kaplan-Meier estimators followed by pair-wise comparisons using the log-rank test (Mantel-Cox).

The effect of medfly genetic background and *Wolbachia* infection on adult sex ratio, fecundity, males’ mating competitiveness and flight ability was determined by two-way ANOVA. The effect of the *Wolbachia* strain on the aforementioned parameters was estimated by one way ANOVA.

The effect of medfly genetic background, *Wolbachia* infection, *Wolbachia* strain and adult sex on longevity under water and food deprivation were determined by Cox regression analysis [42].

In all tests, the level of significance was set at α = 0.05.

## Results

### *Wolbachia* status of the five laboratory strains

All flies of the three *Wolbachia* infected medfly lines (60 out of 60 individuals) produced the expected 16S *rRNA* gene amplicon, while all screened flies of the two uninfected medfly lines (40 out of 40) were found negative (data not shown). The *wsp* based PCR screening that discriminates among the different *w*Cer *Wolbachia* strains was performed on the same flies of the three *Wolbachia-*infected medfly lines and 56S2 plus 88.6 produced only the expected *w*Cer2-specific *wsp* amplicon (40 out of the 40 flies), while all flies from the S10.3 line produced only the *w*Cer4-specific *wsp* amplicon (data not shown). Three individuals (out of the 20) were selected per *Wolbachia-*infected medfly line and the MLST profile was analyzed through sequencing of the MLST genes. Again, all flies presented the expected MLST profile, as described in the *Wolbachia* MLST database.

### Effects of *Wolbachia* infection on hatch rate

**Fig. 1:**
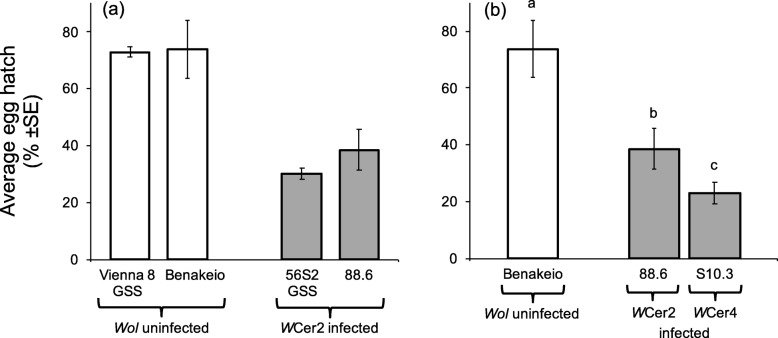
Egg hatch. Effect of (**a**) *Wolbachia* infection and medfly genotype, and (**b**) *Wolbachia* strain on the same medfly genotype on egg hatch rates. White columns represent average percent egg hatch of *Wolbachia* uninfected and grey columns that of *Wolbachia* infected lines. Columns headed with different letter are significantly different (*P* < 0.05) (sample sizes: *Vienna 8: 4036, 56S2 eggs: 2984, Benakeio: 2886 eggs, 88.6: 2899 eggs, S10.3: 2934* eggs)

Logistic regression analysis revealed that both the genetic background of medfly and the *Wolbachia* infection, as well as their interaction were significant predictors of egg hatch (Wald’s t-test =55.68, df = 1, *P* < 0.0001; Wald’s t-test = 782.96, df = 1, *P* < 0.0001, and Wald’s t-test = 7.39, df = 1, *P* = 0.007, respectively). As shown in Fig. 1a, in both medfly genetic backgrounds, *Wolbachia* infection detrimentally reduced female fertility. Different *Wolbachia* strains exerted differential reduction in egg hatch rates on the same medfly genetic background (x^2^ = 1757.49, df = 2, *P* < 0.001). Chi-square test revealed significant differences between the infected 88.6 and S10.3 lines, and the uninfected Benakeio line (x^2^ = 1757.49, df = 2, *P* < 0.001). Both *Wolbachia* strains (*w*Cer2 or *w*Cer4) reduced hatch rates compared to the uninfected flies (x^2^ = 833.37 and 1666.67, df = 1, *P* < 0.001). Hatch rates were lower in *w*Cer4 than in *w*Cer2 infected lines (x^2^ = 174.72, df = 1, *P* < 0.0001) (Fig. 1b).

### Effects of *Wolbachia* infection on larval and pupal survival

**Fig. 2:**
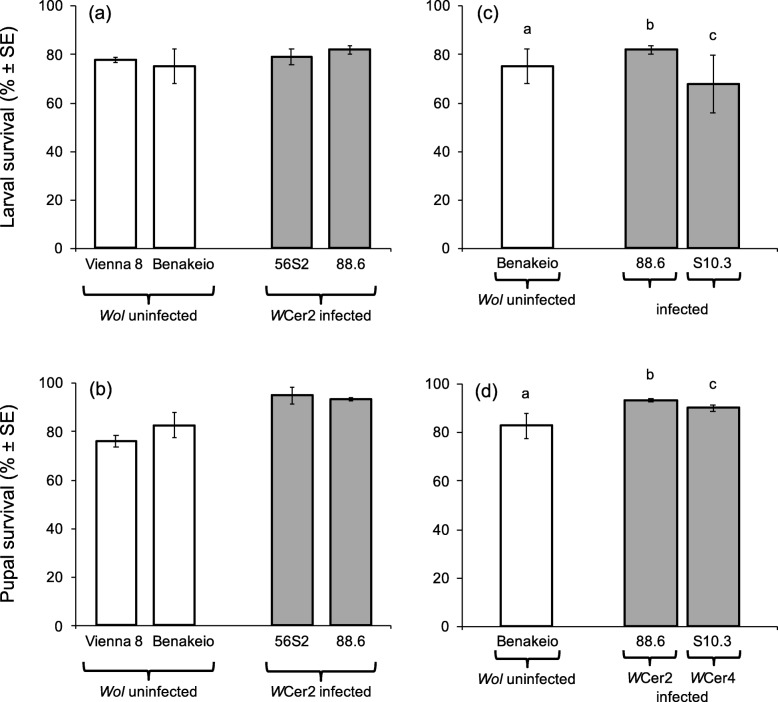
Larval and pupal survival. Effect of *Wolbachia* infection and medfly genotype (**a**, **b**), and *Wolbachia* strain on the same medfly genotype (**c**, **d**), on percent survival of immature stages [larval (**a**, **c**) and pupal (**b**, **d**)]. White columns represent percent survival of *Wolbachia* uninfected immature medflies and grey columns that of *Wolbachia* infected lines. Columns headed with different letter are significantly different (*P* < 0.05) (sample sizes: *Vienna 8: 2975 and 2314, 56S2: 913 and 683, Benakeio: 2231 and 1768, 88.6: 1157 and 956, S10.3: 698 and 447, larvae and pupae respectively*)

*Wolbachia* infection was not a significant predictor of larval survival (Wald’s t-test t = 0.521, df = 1, *P* = 0.470) (Fig. 2a). However, it increased survival rates of pupae (Wald’s t-test t = 7.805, df = 1, *P* = 0.005) in both medfly genetic backgrounds (Fig. 2b). The effect of the different medfly genetic backgrounds was also significant predictor of both larval and pupal stage survival (Wald’s t-test t = 11.842, df = 1, *P* = 0.001 and Wald’s t-test t = 48.016, df = 1, *P* < 0.001, respectively). The interaction between *Wolbachia* infection and medfly genetic background was a significant predictor of pupal survival indicating a differential response of the two medfly genotypes (Wald’s t-test t = 17.386, df = 1, *P* < 0.001) (Fig. 2a). As far as effects of different *Wolbachia* strains on survival during larval and pupal stages are regarded, chi-square test revealed significant differences between the 88.6 and S10.3 lines, and the uninfected Benakeio line (x^2^ = 94.159 and 25.642, df = 2, *P* < 0.0001) (Figs. 2c, d). The *Wolbachia* strain *w*Cer2 increased both larval and pupal survival compared to the uninfected flies (x^2^ = 5.525, df = 1, *P* = 0.019 and x^2^ = 7.948, df = 1, *P* = 0.005). Conversely, the *Wolbachia* strain *w*Cer4 reduced the survival in the larval stage whereas it increased the survival in the pupal stage when compared to the uninfected lines (x^2^ = 66.693, df = 1, *P* < 0.001 and x^2^ = 25.304, df = 1, *P* = 0.001). Both survival during the larval and pupal stage were lower in the *w*Cer4 infected flies compared to wCer2 infected ones (x^2^ = 81.615, df = 1, *P* < 0.001 and x^2^ = 5.274, df = 1, *P* = 0.021, respectively) (Figs. 2c, d).

### Effects of *Wolbachia* infection on immature development

**Fig. 3:**
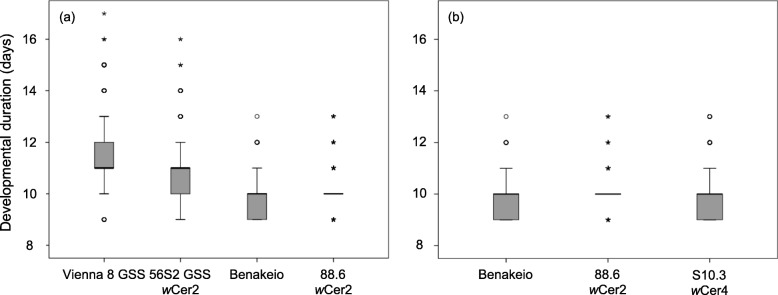
Duration of Immature stage developmental period. Box-plot diagram showing the effect of (**a**) *Wolbachia* infection and medfly genotype, and (**b**) *Wolbachia* strain on the same medfly genotype, on immature developmental duration in days (egg to pupa)

The effect of *Wolbachia* infection on the embryonic and larval developmental duration of the five medfly laboratory lines is depicted in Fig. 3. Cox regression analysis revealed that both the genetic background of medfly and *Wolbachia* infection were significant predictors of egg to pupae developmental duration (Wald’s t-test = 290.51 and 30.12, df = 1, *P* < 0.0001, respectively), as well as their interaction (Wald’s t-test = 9.36, df = 1, *P* < 0.0001). The infection reduced egg to pupae duration on VIENNA 8 GSS genetic background whereas it prolonged the respective duration on the BENAKEIO flies (Fig. 3a).

Survival analysis revealed significant differences in egg to pupae duration as well among uninfected Benakeio, and the infected 88.6 and S10.3 lines (log rank test: x^2^ = 82.19, *P* < 0.0001). Specifically, *Wolbachia* infection, either *w*Cer2 or *w*Cer4, prolonged the egg and larval developmental duration when compared to the uninfected flies (x^2^ = 74.115, 38.014: *P* < 0.0001for 88.6 and S10.3, respectively). No differences were found between the *Wolbachia* infected lines (x^2^ = 0.102, *P* = 0.750), (Fig. 3b).

**Fig. 4:**
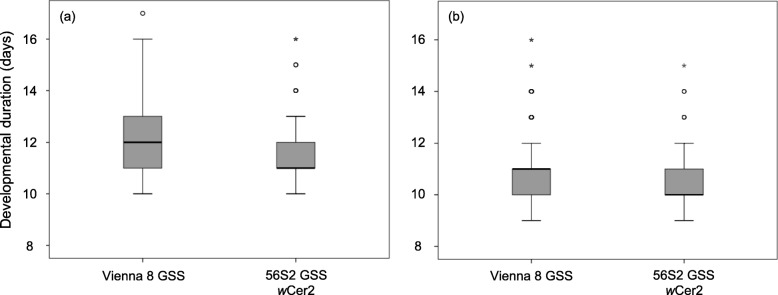
Immature stages development on the Genetic Sexing Strains (GSS). Box-plots showing the effect of *Wolbachia* infection on immature developmental duration in days (egg to pupa) of the VIENNA 8 GSS (**a**) females, and (**b**) males

Cox regression analysis revealed that both *Wolbachia* infection and sex were significant predictors of the pre-pupa duration when the GSS lines (*Wolbachia* infected 56S2 GSS and the uninfected Vienna 8 GSS) were compared (Wald’s t-test = 55.58 and 99.11, df = 1, *P* < 0.0001, respectively). The interaction between *Wolbachia* infection and sex was not significant (Wald’s t-test = 0.88, df = 1, *P* = 0.348) indicating that the bacterium affected the developmental duration of both sexes similarly in the VIENNA 8 GSS genetic background, (Fig. 4).

### Effects of *Wolbachia* infection on adult sex ratio

Two-way ANOVA revealed that neither the genetic background of medfly (F = 0.046, df = 1,9, *P* = 0.835) nor *Wolbachia* infection (F = 0.793, df = 1,9, *P* = 0.396) and their interaction (F = 0.010, df = 1,9, *P* = 0.924) were significant predictors of the sex ratio (male/female) of the emerged adults. Likewise, the *Wolbachia* strain (*w*Cer2 and *w*Cer4) was not a significant predictor of the sex ratio of emerged adults (F = 0.073, df = 2,6, *P* = 0.931; see Additional file 2).

### Effects of *Wolbachia* infection on adults’ longevity

**Fig. 5:**
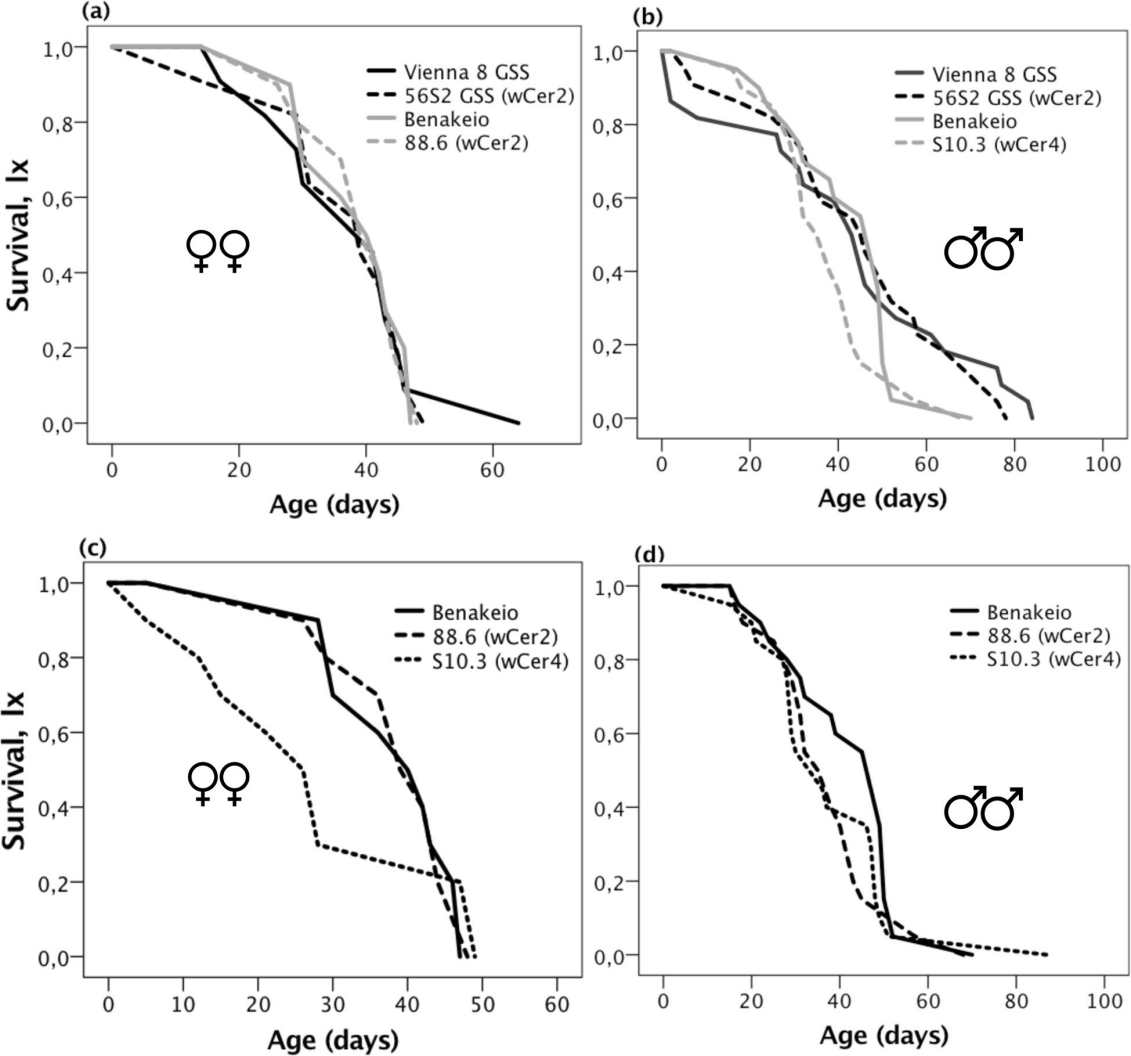
Adult longevity. Survival curves (l_x_) showing the effects of *Wolbachia* infection and medfly genotype (**a**-**b**), and *Wolbachia* strain on the same medfly genotype (**c**-**d**), on females and males longevity

Neither *Wolbachia* infection nor medfly genetic background were significant predictors of adult lifespan (Wald’s t-test = 1.07 and 3.75, df = 1, *P* = 0.300 and 0.053, respectively) in contrast, sex was significant as males outlived females (Wald’s t-test = 6.491, df = 1, *P* = 0.011), (Figs. 5a, b). Neither *Wolbachia* strain nor sex were significant predictors of adult longevity on the BENAKEIO flies (Wald’s t-test = 1.65 and 3.47, df = 1, *P* = 0.199 and 0.062, respectively), (Fig. 5c, d). The *w*Cer2 infected females suffered reduced survival rates compared to uninfected and the *w*Cer4 infected ones, but this observation was not significant (Fig. 5c).

### Effects of *Wolbachia* infection on fecundity

**Fig. 6:**
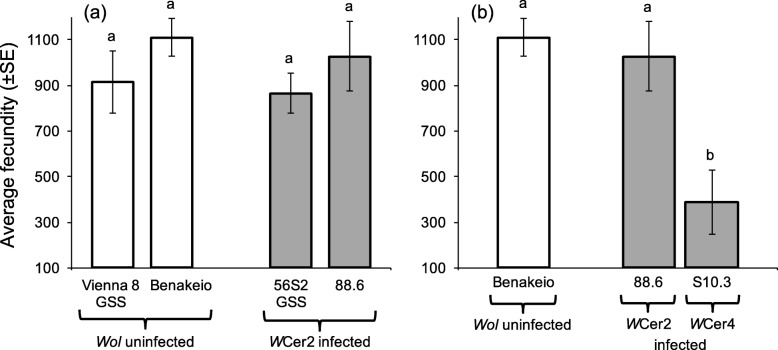
Fecundity rates. Effect of (**a**) *Wolbachia* infection and medfly genotype, and (**b**) *Wolbachia* strain on the same medfly genotype, on females’ egg production. White columns represent average fecundity of *Wolbachia* uninfected and grey columns that of *Wolbachia* infected lines. Columns headed with the same letter are not statistically significantly different (Tukey’s HSD test, *P* > 0.05)

Two-way ANOVA [on ln(x) transformed lifetime fecundity rates to achieve normality and equal variance] revealed that neither the medfly genetic background (F = 2.388, df = 1,38, *P* = 0.131) nor *Wolbachia* infection (F = 0.310, df = 1,38, *P* = 0.581) affected lifetime fecundity rates. Similarly, the interaction between medfly genetic background and *Wolbachia* infection was not significant as well (F = 0.367, df = 1,38, *P* = 0.548). In contrast, fecundity rates were significantly different among *Wolbachia* infected medfly lines S10.3, and both the 88.6 and uninfected BENAKEIO (F = 9.451, df = 2,28, *P* = 0.001) (Fig. 6).

### Effects of *Wolbachia* infection on male mating competitiveness

**Fig. 7:**
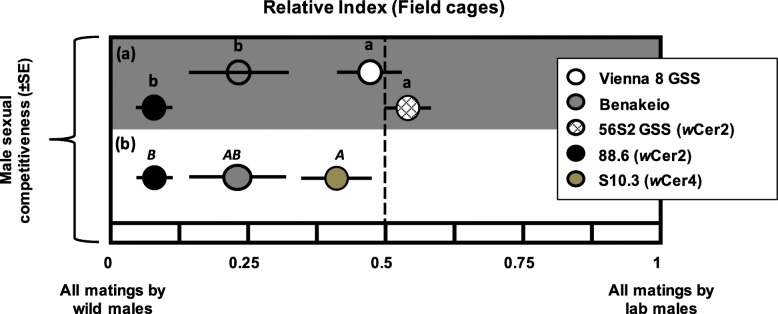
Males sexual competitiveness. Effect of (**a**) *Wolbachia* infection and medfly genotype, and (**b**) *Wolbachia* strain on the same medfly genotype, on male sexual competitiveness (marks represent the Relative Index values). Marks indicated with the same letter in each graph are not statistically significantly different (Tukey’s HSD test, *P* > 0.05)

We used the RI index (Relative Index, analogous to the Relative Sterility Index (RSI), see FAO/IAEA/USDA 2014) to compare mating competitiveness of males of the five medfly lines tested against wild males in competition for wild females (Fig. 7). Overall, *Wolbachia* infection did not affect mating competitiveness (F = 0.553, df = 1,21, *P* = 0.465) in contrast to medfly genetic background (F = 45.849, df = 1,21, *P* < 0.0001). The interaction between medfly genetic background and *Wolbachia* infection was marginally significant (F = 4.636, df = 1,21, *P* = 0.043) indicating a rather differential impact of the *Wolbachia* infection on the two different medfly genetic backgrounds (Fig. 7a). One way ANOVA revealed significant differences in male mating competitiveness among BENAKEIO uninfected, S10.3 and 88.6 lines (F = 9.450, df = 1,12, *P* = 0.003). *w*Cer2 and *w*Cer4 infections reduced and increased male mating competitiveness, respectively (Fig. 7b).

### Effects of *Wolbachia* infection on flight ability

**Fig. 8:**
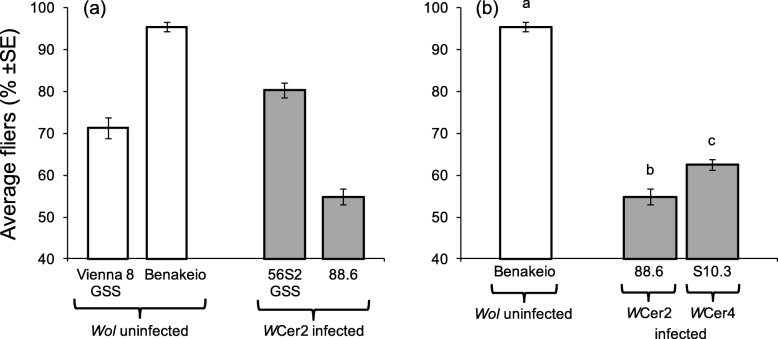
Adults flight ability. Effect of (**a**) *Wolbachia* infection and medfly genotype, and (**b**) *Wolbachia* strain on the same medfly genotype, on flight ability. White columns represent average percent fliers of *Wolbachia* uninfected and grey columns that of *Wolbachia* infected lines. Columns headed with different letter are significantly different (Tukey’s HSD test, *P* < 0.05)

*Wolbachia* infection was a significant predictor of adult flight ability (F = 70.42, df = 1,16, *P* < 0.0001), in contrast to the medfly genetic background (F = 0.10, df = 1,16, *P* = 0.754). The significant interaction between *Wolbachia* infection and medfly genetic background highlights the differential effect of *Wolbachia* infection on the two medfly genetic backgrounds resulting in positive and negative effects on flight performance on VIENNA 8 GSS and BENAKEIO flies respectively (F = 173.49, df = 1,16, *P* < 0.0001) (Fig. 8a).

*Wolbachia* infection, regardless of the bacteria strain, significantly reduced the flight ability of the BENAKEIO flies (F = 216.34, df = 2,12, *P* < 0.0001). Significant differences between the two infected lines were also observed (*P* < 0.05) (Fig. 8b).

**Fig. 9:**
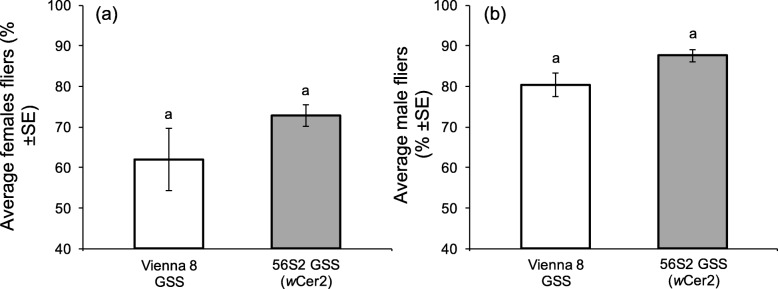
Adults flight ability on the Genetic Sexing Strains (GSS). Effect of *Wolbachia* infection on the flight ability of VIENNA 8 GSS (**a**) females, and (**b**) males. White columns represent average percent fliers of the *Wolbachia* uninfected and grey columns that of *Wolbachia* infected line. Columns headed with the same letter are not significantly different (Tukey’s HSD test, *P* > 0.05)

Considering only the VIENNA 8 GSS medfly genetic background, two way ANOVA revealed that neither *Wolbachia* infection nor sex were significant predictors of the flight ability (F = 25.00, df = 1,16, *P* = 0.126 and F = 85.05, df = 1,16, *P* = 0.069, respectively). Similarly, the interaction between *Wolbachia* infection and adult sex was not significant (F = 0.172, df = 1,16, *P* = 0.684), (Fig. 9).

### Effects of *Wolbachia* infection on longevity under food and water deprivation

**Fig 10:**
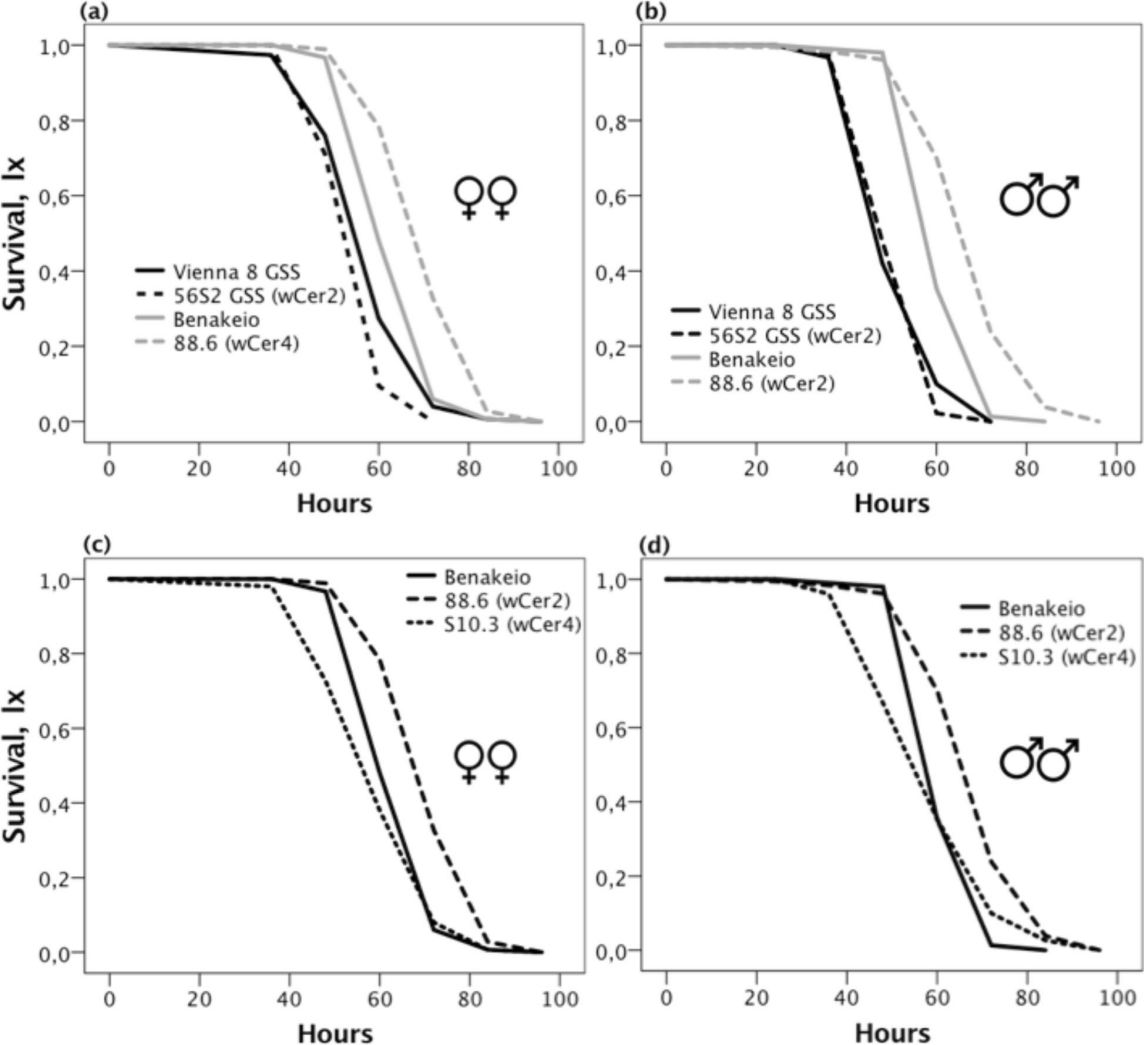
Adult survival under stress conditions. Survival curves (l_x_) showing the effect of *Wolbachia* infection and medfly genotype (**a**-**b**), and *Wolbachia* strain on the same medfly genotype (**c**-**d**), on females and males survival under food and water deprivation

Medfly genetic background, *Wolbachia* infection and sex were significant predictors of adult longevity under food and water deprivation (Wald’s t-test = 224.17, 37.28 and 30.25, df = 1, *P* < 0.0001). The significant interaction between medfly genetic background and *Wolbachia* infection (Wald’s t-test = 39.72, df = 1, *P* < 0.0001) highlights the differential effect of the *Wolbachia* infection on the two medfly lines. Specifically, *Wolbachia* infection reduced VIENNA 8 GSS longevity under water and food deprivation, whereas it increased the BENAKEIO longevity under the same stress conditions (Fig. 10a, b).

For the BENAKEIO flies, Cox regression analysis revealed that the *Wolbachia* strain significantly affected adult longevity under water and food deprivation (Wald’s t-test = 62.01, df = 2, *P* < 0.0001). Nevertheless, neither sex (Wald’s t-test = 2.26, df = 1, *P* = 0.133) nor the interaction between the different medfly lines (BENAKEIO genetic background) and sex were significant predictors of adult longevity under food and water deprivation (*P* > 0.05). Adults of the uninfected Benakeio showed similar stress tolerance compared to S10.3 (Wald’s t-test = 1.58, df = 1, *P* = 0.208) but much lower compared to the *w*Cer2 infected ones (Wald’s t-test = 35.15, df = 1, *P* < 0.0001). Adults of the 88.6 line expressed longer survival rates under food and water deprivation compared to S10.3 ones (Wald’s t-test = 54.13, df = 1, *P* < 0.0001), (Fig. 10c, d).

Focusing on the VIENNA 8 GSS medfly genetic background, statistical analysis showed that *Wolbachia* infection was not a significant predictor of longevity under stress (Wald’s t-test = 2.734, df = 1, *P* = 0.098), in contrast to sex (Wald’s t-test = 22.52, df = 1, *P* < 0.0001) (Fig. 10a, b).

## Discussion

Our results demonstrate that *Wolbachia* infection modifies several fitness components of mass-reared Mediterranean fruit flies. The outcome of the effects seems to be regulated both by medfly genetic background and *Wolbachia* strain resulting in a complex range of outputs. *Wolbachia* infection reduces fertility rates in both medfly genetic backgrounds and shortens the pre-pupa developmental duration in the GSS strain. On the other hand, regardless of the strain, *Wolbachia* infection does not affect either the sex ratio or the longevity of adults. *w*Cer4 infection imposed a reduction in females’ fecundity but *w*Cer2 did not. Males mating competitiveness, adults flight ability and longevity under water and food deprivation were affected by both medfly genetic background of medfly and the strain of *Wolbachia* (genotype by genotype interaction).

### Effects on medfly life-history traits

Similar to earlier studies, our results point out a detrimental effect of *Wolbachia* infection on fertility in both medfly genetic backgrounds [33–35, 43, 44]. Embryonic mortality ranged from 50 to 60% and the effect of the *w*Cer4*Wolbachia* strain was more pronounced when compared to *w*Cer2. The different effects of *Wolbachia* strains on hatch rates have also been reported in mosquitoes [43, 44]. Our results are consistent with previous reports, which also mention a fertility advantage of *w*Cer2 over *w*Cer4 [33–35]. The amount of sperm transferred and the secretions of male accessory glands could both affect medfly female fertility [45]. *Wolbachia* infected *Drosophila simulans* males produce lower sperm quantities and transfer fewer sperm compared to uninfected ones resulting in lower fertility rates [46, 47]. Whether the reduced hatch rates reported here are the result of lower sperm production or sperm transfer because of the *Wolbachia* infection needs to be explored in future studies.

*Wolbachia* infection reduced the immature developmental duration in the Vienna 8 GSS line, whereas it seems to prolong the developmental duration in the BENAKEIO line. Working with the same medfly genotypes almost 8 years ago, Sarakatsanou et al. (2011) [35] reported that *Wolbachia* shortened the developmental duration of immature stages in both the VIENNA 8 and BENAKEIO flies. Considering that the transinfection into VIENNA 8 GSS genotype is more recent than into BENAKEIO genotype [33, 34] the differences recorded in the two studies might reflect the dynamic nature of the symbiotic interactions between medfly and *Wolbachia* as was also shown in the case of *Drosophila simulans* [16]. Moreover, it must be pointed out that the two studies were conducted under different rearing protocols, which could also affect the outcome of these effects. Recent studies demonstrated that the *Wolbachia* titer in *Drosophila melanogaster* and *D. simulans* could be nutrient-dependent, and therefore different diets may differentially modify biological traits [48]. In addition, applying different rearing methods in a given insect population could lead to continuous selection for specific characteristics resulting in the establishment of distinct laboratory colonies with slightly different biological traits [49]. Reynolds et al. (2003) [50] recorded that *Wolbachia* prolongs the developmental duration of immature stages in *D. melanogaster,* whereas Poinsot et al. (1997) [51] did not detect any effect on *D. simulans*. Comparing three *Wolbachia-*infected lines in the same *Aedes albopictus* genomic background, Zhang et al. (2015) [30] found that *w*Pip accelerated immature development, whereas Islam and Dobson (2006) [52] also reported differences in developmental rates among an uninfected, a single and a superinfected *Ae. albopictus* line*.* On the other hand, *Wolbachia* infection did not affect the developmental rates of *Aedes aegypti* and *Anopheles stephensi* [20, 53].

The work of Sarakatsanou et al. (2011) [35] demonstrated that *Wolbachia* imposes a significant reduction on *C. capitata* fecundity and adult longevity. In the current study we found that the effect of *Wolbachia* on medfly fecundity is strain-specific since *w*Cer4 and *w*Cer2 infection induced negative and neutral effects on egg production respectively. Apart from *C. capitata,* many reports suggest that *Wolbachia* could elicit positive, negative or neutral response on hosts fecundity and life span indicating that *Wolbachia* effects could vary among different insect species, strains or even sexes within species [8, 11–15, 17–20, 29–31, 54, 55].

### Effects on flight ability, response to food and water deprivation and male mating competitiveness

Medfly mating competitiveness against wild males for wild females is determined both by medfly genetic background of medfly and the *Wolbachia* strain. *Wolbachia* infection did not affect the performance of males of the VIENNA 8 GSS, which is the currently used medfly line in most of the SIT large scale operational programs. Previous studies on female preference for mating partners in other insect taxa (Drosophilae and Culicidae) revealed both positive and negative effects of *Wolbachia* infection. *Wolbachia* infected *D. simulans* and *D. melanogaster* males showed higher mating rates (number of copulations) compared to uninfected ones when a mixed population of infected and uninfected females were offered as mating partners [56]. Similar studies in mosquitoes revealed that *Wolbachia-*infected males could compete effectively with wild males of *Ae. aegypti* [57], *Aedes polynesiensis* [58] and *Ae. albopictus* [31, 59]. On the other hand, *Wolbachia* infected *Anopheles stephensi* males are less competitive against uninfected ones for mating [20]. Large-scale field studies should be conducted in order to fully elucidate effects of *Wolbachia* in medfly males.

We also investigated the effect of *Wolbachia* on flight ability (an index of locomotor activity) and adult longevity under food and water deprivation. *Wolbachia* infection increased the number of fliers on the VIENNA 8 GSS, whereas reduced the number of fliers on BENAKEIO lines. Two previous studies assessed the impact of *Wolbachia* infection on insects’ locomotor activity. *Wolbachia*-infected *Aedes aegypti* adult mosquitoes displayed increased locomotor activity compared to uninfected ones [60]. On the other hand, Dedeine et al. (2001) [61] following similar experimental procedure did not detect significant effects of *Wolbachia* infection on the locomotor activity of the parasitic wasp *Asobara tabida*. As far as medfly response under stress conditions is concerned, our results showed that *Wolbachia* infection reduced the longevity under water and food deprivation in VIENNA 8 GSS females while it did not exert any effect on VIENNA 8 GSS males. On the other hand, by testing the BENAKEIO genotype, we recorded that the *w*Cer2 infection confers a significant improvement in longevity under the given stress conditions to both sexes. To the best of our knowledge, there are no previous data available regarding the ability of *Wolbachia* infected arthropods to survive under certain stress conditions.

## Conclusions

In the present study we evaluated several fitness parameters of medflies comparing the same insect genotype under the presence and absence of *Wolbachia*. As previously noted, this is the safer path in a trial to detect ultimate benefits or detrimental effects of *Wolbachia* infection*.* This is because such an experimental approach minimizes the possibility to attribute effects caused by other factors to *Wolbachia* infection (e.g. curing the infection with antibiotic is a popular but questionable practice in fitness related studies) [62]. Our findings highlight the determinant role of the genotypes (insect host and *Wolbachia*) interaction in the expression of specific phenotypes and the potential inconsistency of certain fitness parameters over the symbiosis historic “time-line”. In general, our data reveal that *Wolbachia* infection could alter important life history traits of mass-reared *C. capitata* lines. The response of each genotype to *Wolbachia* infection should be considered toward ensuring the productivity of *Wolbachia*-infected insects under mass-rearing conditions.

*Wolbachia* symbiosis could be a promising tool in support of population suppression of insect pests of agricultural, veterinary and human health importance. However, this will first require the evaluation of the potential impact *Wolbachia* infection may have on key life history traits and particularly on those affecting rearing efficiency and male mating competitiveness of an insect line candidate for SIT and/or IIT applications. Appropriate models should also be developed including cost benefit analysis which will determine their suitability for large scale operational programs.

## Supplementary information

**Additional file 1.** Biological material used in the experiments.

**Additional file 2. **Adult sex ratio. Effect of (a) *Wolbachia* infection and medfly genotype, and (b) *Wolbachia* strain on the same medfly genotype, on adult sex ratio. White columns represent the average fraction [number of males]/[number of females] of *Wolbachia* uninfected and grey columns that of *Wolbachia* infected lines. Columns headed with the same letter are not significantly different (Tukey’s HSD test, *P* > 0.05).

## Data Availability

The datasets used and analyzed during the current study are available from the corresponding author on reasonable request.

## Abbreviations

AW-IPM: Area Wide Integrated Pest Management
FAO: Food and Agriculture Organization
GSS: Genetic Sexing Strain
IAEA: International Atomic Energy Agency
IIT: Incompatible Insect Technique
IPCL: Insect Pest Control Laboratory
SIT: Sterile Insect Technique
USDA: United States Department of Agriculture

## References

[1] Hertig M (1936). The rickettsia, *Wolbachia pipientis* (gen. et sp. n.) and associated inclusions of the mosquito, *Culex pipiens*. Parasitology.

[2] Zug R, Hammerstein P. Still a host of hosts for *Wolbachia*: Analysis of recent data suggests that 40% of terrestrial arthropod species are infected. PLoS One. 2012;7 Available from: wos:000305351700044. 10.1371/journal.pone.0038544 PMC3369835 22685581

[3] Hoffmann AA, Turelli M. Cytoplasmic incompatibility in insects. Influ Passengers. 1997:42–80 Available from: ccc:000073362000002.

[4] O’Neill SL, Hoffmann AA, Werren JH (1997). Influential passengers: inherited microorganisms and arthropod reproduction.

[5] Stouthamer R., Breeuwer J. A. J., Hurst G. D. D. (1999). Wolbachia Pipientis: Microbial Manipulator of Arthropod Reproduction. Annual Review of Microbiology.

[6] Yen JH, Barr AR (1971). New Hypothesis of cause of cytoplasmic incompatibility in *Culex pipiens* l. Nature.

[7] Boller EF, Russ K, Vallo V, Bush GL (1976). Incompatible races of European cherry fruit-fly, *Rhagoletis cerasi* (Diptera-Tephritidae), Their origin and potential use in biological control. Entomol Exp Appl.

[8] Hoffmann AA, Turelli M, Harshman LG (1990). Factors affecting the distribution of cytoplasmic incompatibility in *Drosophila simulans*. Genetics.

[9] Stouthamer R., Breeuwer J. A. J., Luck R. F., Werren J. H. (1993). Molecular identification of microorganisms associated with parthenogenesis. Nature.

[10] Stolk C, Stouthamer R (1996). Influence of a cytoplasmic incompatibility-inducing *Wolbachia* on the fitness of the parasitoid wasp *Nasonia vitripennis*. Proc Sect Exp Appl Entomol.

[11] Min KT, Benzer S (1997). *Wolbachia*, normally a symbiont of *Drosophila*, can be virulent, causing degeneration and early death. Proc Natl Acad Sci U S A.

[12] Dobson SL, Fox CW, Jiggins FM (2002). The effect of *Wolbachia*-induced cytoplasmic incompatibility on host population size in natural and manipulated systems. Proc R Soc B Biological Sci.

[13] Mcgraw EA, Merritt DJ, Droller JN, O’Neill SL (2002). *Wolbachia* density and virulence attenuation after transfer into a novel host. Proc Natl Acad Sci U S A.

[14] Fry AJ, Palmer MR, Rand DM (2004). Variable fitness effects of *Wolbachia* infection in *Drosophila melanogaster*. Heredity.

[15] Riegler M, Charlat S, Stauffer C, Mercot H (2004). *Wolbachia* transfer from *Rhagoletis cerasi* to *Drosophila simulans*: Investigating the outcomes of host-symbiont coevolution. Appl Environ Microbiol.

[16] Weeks AR, Turelli M, Harcombe WR, Reynolds K, Hoffmann AA. From parasite to mutualist: Rapid evolution of *Wolbachia* in natural populations of *Drosophila*. Plos Biol. 2007:997–1005 Available from: wos:000246716700008. 10.1371/journal.pbio.0050114 PMC1852586 17439303

[17] McMeniman CJ, Lane RV, Cass BN, Fong AW, Sidhu M, Wang YF (2009). Stable Introduction of a Life-shortening *Wolbachia* infection into the mosquito *Aedes aegypti*. Science.

[18] Suh E, Mercer DR, Fu Y, Dobson SL (2009). Pathogenicity of Life-shortening *Wolbachia* in *Aedes albopictus* after transfer from *Drosophila melanogaster*. Appl Environ Microbiol.

[19] Miller WJ, Ehrman L, Schneider D. Infectious Speciation revisited: Impact of symbiont-depletion on female fitness and mating behavior of *Drosophila paulistorum*. Plos Pathog. 2010; Available from: wos:000285587500004. 10.1371/journal.ppat.1001214 PMC2996333 21151959

[20] Joshi Deepak, McFadden Michael J, Bevins David, Zhang Fengrui, Xi Zhiyong (2014). Wolbachia strain wAlbB confers both fitness costs and benefit on Anopheles stephensi. Parasites & Vectors.

[21] Koukou Katerina, Pavlikaki Haris, Kilias George, Werren John H., Bourtzis Kostas, Alahiotis Stamatis N. (2006). INFLUENCE OF ANTIBIOTIC TREATMENT AND WOLBACHIA CURING ON SEXUAL ISOLATION AMONG DROSOPHILA MELANOGASTER CAGE POPULATIONS. Evolution.

[22] Markov VA, Lazebny O, Goryacheva II, Antipin IM, Kulikov A (2009). Symbiotic bacteria affect mating choice in *Drosophila melanogaster*. Anim Behav.

[23] Gazla IN, Carracedo MC (2011). *Wolbachia* induces sexual isolation in *Drosophila melanogaster* and *Drosophila simulans*. Open J Genet.

[24] Liu C, Wang JL, Zheng Y, Xiong EJ, Li JJ, Yuan LL (2014). *Wolbachia*-induced paternal defect in *Drosophila* is likely by interaction with the juvenile hormone pathway. Insect Biochem Mol Biol.

[25] Mitchell WC, Saul SH (1990). Current control methods for the Mediterranean fruit fly, *Ceratitis capitata*, and their application in the USA. Rev Agric Entomol.

[26] Vreysen MJB, Robinson AS, Hendrichs JP (2007). Area-wide control of insect pests: from research to field implementation.

[27] Hendrichs J., Robinson A. S., Cayol J. P., Enkerlin W. (2002). MEDFLY AREAWIDE STERILE INSECT TECHNIQUE PROGRAMMES FOR PREVENTION, SUPPRESSION OR ERADICATION: THE IMPORTANCE OF MATING BEHAVIOR STUDIES. Florida Entomologist.

[28] Mumford JD, Barnes BN (2002). Economic analysis of area-wide fruit fly management.

[29] Zhang D, Zheng X, Xi Z, Bourtzis K, Gilles JRL (2015). Combining the sterile insect technique with the incompatible insect technique: I-impact of *Wolbachia* infection on the fitness of triple-and double-infected strains of *Aedes albopictus*. PLoS One.

[30] Zhang D, Lees RS, Xi Z, Gilles JRL, Bourtzis K (2015). Combining the sterile insect technique with *Wolbachia*-based approaches: II-A safer approach to *Aedes albopictus* population suppression programmes, designed to minimize the consequences of inadvertent female release. PLoS One.

[31] Zhang Dongjing, Lees Rosemary Susan, Xi Zhiyong, Bourtzis Kostas, Gilles Jeremie R. L. (2016). Combining the Sterile Insect Technique with the Incompatible Insect Technique: III-Robust Mating Competitiveness of Irradiated Triple Wolbachia-Infected Aedes albopictus Males under Semi-Field Conditions. PLOS ONE.

[32] Rocha LS, Mascarenhas RO, Perondini ALP, Selivon D. Occurrence of *Wolbachia* in Brazilian samples of *Ceratitis capitata* (Wiedemann) (Diptera : Tephritidae). Neotrop Entomol. 2005:1013, 5 Available from: wos:000234461300020.

[33] Zabalou S., Riegler M., Theodorakopoulou M., Stauffer C., Savakis C., Bourtzis K. (2004). Wolbachia-induced cytoplasmic incompatibility as a means for insect pest population control. Proceedings of the National Academy of Sciences.

[34] Zabalou S., Apostolaki A., Livadaras I., Franz G., Robinson A. S., Savakis C., Bourtzis K. (2009). Incompatible insect technique: incompatible males from aCeratitis capitatagenetic sexing strain. Entomologia Experimentalis et Applicata.

[35] Sarakatsanou A., Diamantidis A. D., Papanastasiou S. A., Bourtzis K., Papadopoulos N. T. (2011). Effects of Wolbachia on fitness of the Mediterranean fruit fly (Diptera: Tephritidae). Journal of Applied Entomology.

[36] Franz G. Genetic sexing strains in Mediterranean fruit fly, an example for other species amenable to large-scale rearing for the sterile insect technique. Sterile Insect Tech Princ Pract. 2005:427–51 Area-Wide Integr. Pest Manag. Available from: ccc:000237286700016.

[37] Caceres C. Mass rearing of temperature sensitive genetic sexing strains in the Mediterranean fruit fly (*Ceratitis capitata*). Genetica. 2002:107–16 Available from: wos:000178951300010. 10.1023/a:1020967810703 12484530

[38] Tanaka N, Steiner LF, Ohinata K, Okamoto R. Low-cost larval rearing medium for mass production of oriental and mediterranean fruit flies. J Econ Entomol. 1969:967 Available from: wos: A1969D934300081.

[39] Werren JH, Windsor DM. *Wolbachia* infection frequencies in insects: evidence of a global equilibrium? Proc R Soc B Biological Sci. 2000:1277–85 Available from: wos:000088222200003. 10.1098/rspb.2000.1139 PMC1690679 10972121

[40] ARTHOFER WOLFGANG, RIEGLER MARKUS, SCHNEIDER DANIELA, KRAMMER MARTIN, MILLER WOLFGANG J., STAUFFER CHRISTIAN (2009). HiddenWolbachiadiversity in field populations of the European cherry fruit fly,Rhagoletis cerasi(Diptera, Tephritidae). Molecular Ecology.

[41] FAO/IAEA/USDA, editor. Product Quality Control for Sterile Mass-Reared and Released Tephritid Fruit Flies, Version 6.0. Vienna: International Atomic Energy Agency, Vienna, Austria; 2014. Available: http://www-naweb.iaea.org/nafa/ipc/public/sterile-mass-reared-v6.pdf.

[42] Collett D (2003). Modelling survival data in medical research.

[43] Xi ZY, Dean JL, Khoo C, Dobson SL (2005). Generation of a novel *Wolbachia* infection in *Aedes albopictus* (Asian tiger mosquito) via embryonic microinjection. Insect Biochem. Mol Biol.

[44] Xi ZY, Khoo CCH, Dobson SL (2005). *Wolbachia* establishment and invasion in an *Aedes aegypti* laboratory population. Science.

[45] Chapman T, Neubaum DM, Wolfner MF, Partridge L (2000). The role of male accessory gland protein Acp36DE in sperm competition in *Drosophila melanogaster*. Proc R Soc B Biological Sci.

[46] Awrahman Z, De Crespigny F, Wedell N (2014). The impact of *Wolbachia*, male age and mating history on cytoplasmic incompatibility and sperm transfer in *Drosophila simulans*. J Evol Biol.

[47] Snook RR, Cleland SY, Wolfner MF, Karr TL (2000). Offsetting effects of *Wolbachia* infection and heat shock on sperm production in *Drosophila simulans*: Analyses of fecundity, fertility and accessory gland proteins. Genetics.

[48] Serbus Laura R., White Pamela M., Silva Jessica Pintado, Rabe Amanda, Teixeira Luis, Albertson Roger, Sullivan William (2015). Correction: The Impact of Host Diet on Wolbachia Titer in Drosophila. PLOS Pathogens.

[49] Parker AG, Dyck VA, Hendrichs J, Robinson AS (2005). Mass-rearing for sterile insect release.

[50] Reynolds KT, Thomson LJ, Hoffmann AA. The effects of host age, host nuclear background and temperature on phenotypic effects of the virulent *Wolbachia* strain popcorn in *Drosophila melanogaster*. Genetics, 2003;164:1027–34 Available from: wos:000184487400016. 10.1093/genetics/164.3.1027 PMC1462616 12871912

[51] Poinsot D, Mercot H (1997). *Wolbachia* infection in *Drosophila simulans*: Does the female host bear a physiological cost?. Evolution.

[52] Islam MS, Dobson SL (2006). *Wolbachia* effects on *Aedes albopictus* (Diptera: Culicidae) immature survivorship and development. J Med Entomol.

[53] Ross Perran A., Yeap Heng Lin, Hoffmann Ary A., Endersby Nancy M. (2014). Larval Competition Extends Developmental Time and Decreases Adult Size of wMelPop Wolbachia-Infected Aedes aegypti. The American Journal of Tropical Medicine and Hygiene.

[54] Alexandrov ID, Alexandrova MV, Goryacheva II, Roshchina NV, Shaikevich EV, Zakharov IA (2007). Elimination of endosymbiont *Wolbachia* specifically decreases competitive ability and longevity of females from laboratory strain of *Drosophila melanogaster*. Russ J Genet.

[55] Fast EM, Toomey ME, Panaram K, Desjardins D, Kolaczyk ED, Frydman HM (2011). *Wolbachia* enhance *Drosophila* stem cell proliferation and target the germline stem cell niche. Science.

[56] De Crespigny F, Pitt T, Wedell N (2006). Increased male mating rate in *Drosophila* is associated with *Wolbachia* infection. J Evol Biol.

[57] Segoli M, Stouthamer R, Stouthamer C, Rugman-Jones P, Rosenheim J (2013). The effect of *Wolbachia* on the lifetime reproductive success of its insect host in the field. J Evol Biol.

[58] Chambers EW, Hapairai L, Peel BA, Bossin H, Dobson SL (2011). Male mating competitiveness of a *Wolbachia*-introgressed *Aedes polynesiensis* strain under semi-field conditions. PLoS Negl Trop Dis.

[59] Wiwatanaratanabutr I, Allan S, Linthicum K, Kittayapong P (2010). Strain-specific differences in mating, oviposition, and host-seeking behavior between *Wolbachia*-infected and uninfected *Aedes albopictus*. J Am Mosq Cont Assoc.

[60] Evans O, Caragata EP, McMeniman CJ, Woolfit M, Green DC, Williams CR (2009). Increased locomotor activity and metabolism of *Aedes aegypti* infected with a life-shortening strain of *Wolbachia pipientis*. J Exp Biol.

[61] Dedeine F, Vavre F, Fleury F, Loppin B, Hochberg ME, Bouletreau M (2001). Removing symbiotic *Wolbachia* bacteria specifically inhibits oogenesis in a parasitic wasp. Proc Natl Acad Sci U S A.

[62] Zug R, Hammerstein P (2015). Bad guys turned nice? A critical assessment of *Wolbachia* mutualisms in arthropod hosts. Biol Rev.

