# Supplementary figures and images for: Medfly-Wolbachia symbiosis: genotype x genotype interactions determine host’s life history traits under mass rearing conditions
Source: BMC Biotechnol. 2019 Dec 18;19(Suppl 2):96. doi: 10.1186/s12896-019-0586-7 (PMC6918550; doi:10.1186/s12896-019-0586-7)

Average males/females  
( $\pm$ SE)

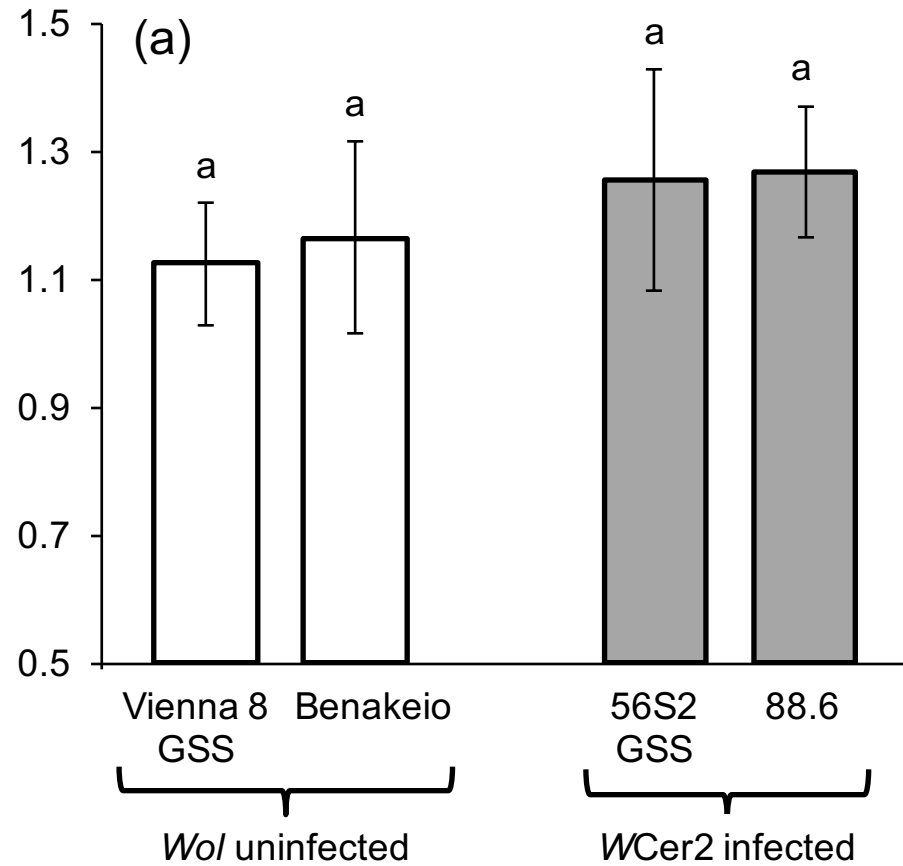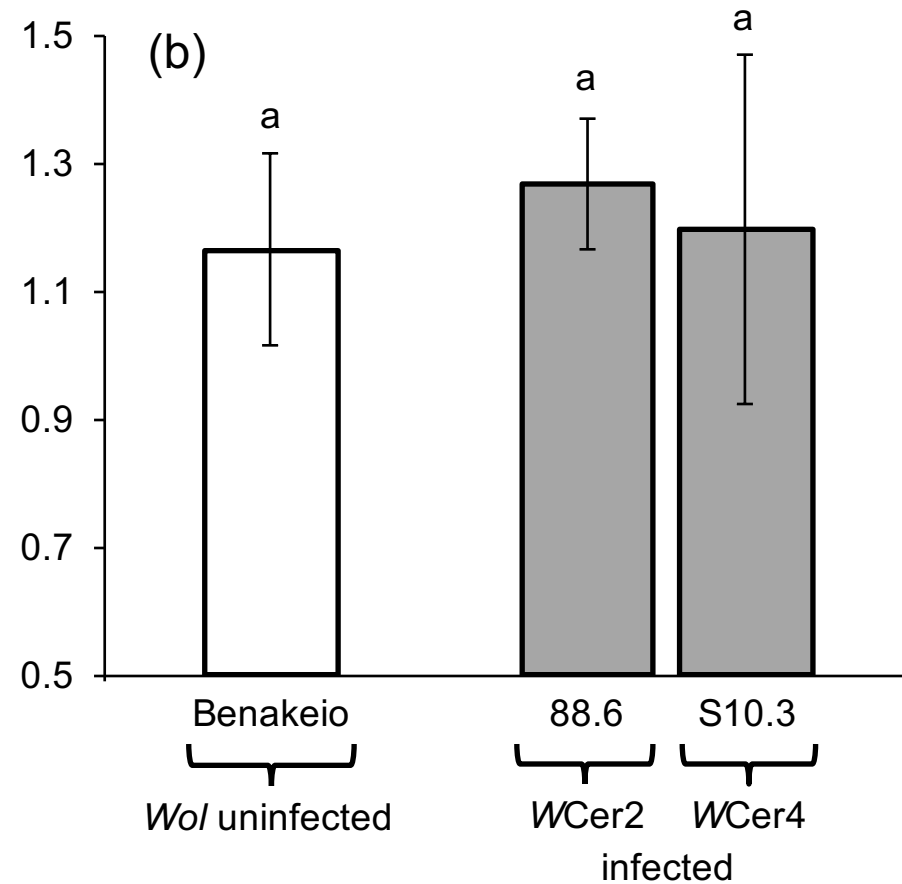

Supplement: Supplementary file 2 — Additional file 2. Adult sex ratio. Effect of (a) Wolbachia infection and medfly genotype, and (b) Wolbachia strain on the same medfly genotype, on adult sex ratio. White columns represent the average fraction [number of males]/[number of females] of Wolbachia uninfected and grey columns that of Wolbachia infected lines. Columns headed with the same letter are not significantly different (Tukey’s HSD test, P > 0.05). [file 12896_2019_586_MOESM2_ESM.pdf]
